# Crossover patterning in plants

**DOI:** 10.1007/s00497-022-00445-4

**Published:** 2022-07-14

**Authors:** Andrew Lloyd

**Affiliations:** Institute of Biological, Environmental & Rural Sciences (IBERS), Aberystwyth University, Penglais, Aberystwyth, SY23 3DA Ceredigion UK

**Keywords:** Crossovers, Recombination, Crossover patterning, Crossover interference

## Abstract

**Key message:**

Chromatin state, and dynamic loading of pro-crossover protein HEI10 at recombination intermediates shape meiotic chromosome patterning in plants.

**Abstract:**

Meiosis is the basis of sexual reproduction, and its basic progression is conserved across eukaryote kingdoms. A key feature of meiosis is the formation of crossovers which result in the reciprocal exchange of segments of maternal and paternal chromosomes. This exchange generates chromosomes with new combinations of alleles, increasing the efficiency of both natural and artificial selection. Crossovers also form a physical link between homologous chromosomes at metaphase I which is critical for accurate chromosome segregation and fertility. The patterning of crossovers along the length of chromosomes is a highly regulated process, and our current understanding of its regulation forms the focus of this review. At the global scale, crossover patterning in plants is largely governed by the classically observed phenomena of crossover interference, crossover homeostasis and the obligatory crossover which regulate the total number of crossovers and their relative spacing. The molecular actors behind these phenomena have long remained obscure, but recent studies in plants implicate *HEI10* and *ZYP1* as key players in their coordination. In addition to these broad forces, a wealth of recent studies has highlighted how genomic and epigenomic features shape crossover formation at both chromosomal and local scales, revealing that crossovers are primarily located in open chromatin associated with gene promoters and terminators with low nucleosome occupancy.

## Introduction

In most sexually reproducing organisms, meiotic crossovers have two key functions. One of these roles is biophysical, ensuring accurate chromosome segregation; crossovers together with sister chromatid cohesion create a physical link between homologous chromosomes during metaphase I, preventing separation of bivalents at the first meiotic division until the spindle assembly checkpoint has been passed. The second major outcome of meiotic crossovers is the reciprocal exchange of segments of homologous chromosomes, generating chromosomes that are a chimaera of maternal and paternal sequence. This reciprocal exchange produces new combinations of alleles and importantly can separate beneficial and detrimental alleles located on the same chromosome. In this way, meiotic recombination speeds adaptation, allowing natural or artificial selection to more efficiently sort beneficial from deleterious mutations (McDonald et al. 2016). The number and location of meiotic crossovers are highly regulated, and at a global scale, this patterning in plants is largely driven by the processes of crossover interference, crossover homeostasis and formation of the obligatory crossover. Despite their importance in determining patterns of inheritance, the molecular basis of all three of these processes has remained largely obscure. Recent studies in plants, however, have shed new light on these classically observed phenomena and suggest that HEI10 (Ziolkowski et al. 2017; Morgan et al. 2021) and ZYP1 (Capilla-Pérez et al. 2021; France et al. 2021) may be key players. This review will provide an overview of these recent studies and consider their implications on crossover interference, crossover homeostasis and formation of the obligatory crossover. In addition, this review will summarise recent genomic analyses in both Arabidopsis and crops which have uncovered numerous genomic and epigenomic factors shaping crossover patterning at both chromosomal and local scales. These studies highlight that crossovers are primarily located in open chromatin with low levels of DNA methylation and are particularly enriched in gene promoters and terminators.

## Meiotic recombination pathways

**Fig. 1:**
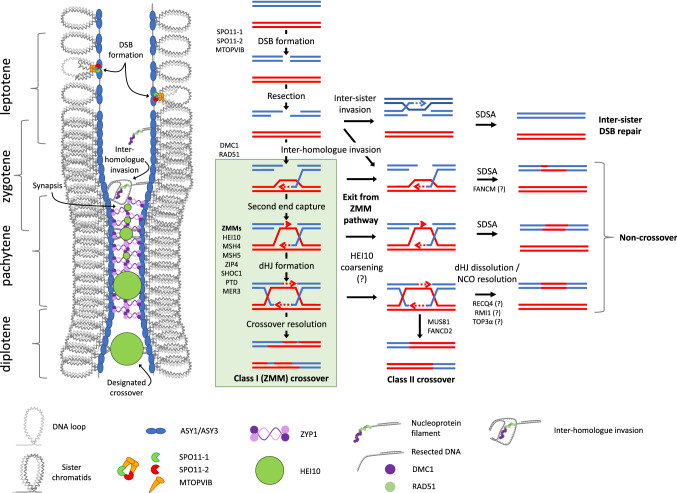
Model of meiotic recombination/DNA repair pathways in plants. Meiosis begins with the assembly of a proteinaceous axis (incorporating ASY1 and ASY3), which forms the base of chromatin loops, and the programmed induction of DNA double-strand breaks by SPO11-1, SPO11-2 and MTOPVIB. Following DSB induction, DNA ends are resected, and the single-stranded overhangs bound by DMC1 and RAD51 to mediate inter-homologue strand-invasion/D-loop formation. These joint molecules are then processed by numerous parallel pathways to be resolved as crossovers or non-crossovers. DSBs can also be repaired without inter-homologue invasion via the sister chromatid. While hundreds of DSBs are induced at the beginning of meiosis, only a small percentage become crossovers. Many recombination intermediates initially enter the class I (ZMM) crossover pathway and have early association with ZMM proteins including HEI10. However, most intermediates exit this pathway and lose their association with HEI10 (HEI10 coarsening) so that by late pachytene, only the few intermediates destined to become class I crossovers remain. These class I crossovers account for ~ 70–90% of crossovers in plants. Joint-molecules/recombination intermediates not destined to become class I crossovers have several potential fates. Helicases (FANCM, RECQ4) and associated proteins disassemble recombination intermediates and promote non-crossover formation via synthesis dependent strand annealing (SDSA) or double Holiday junction (dHJ) dissolution. Other intermediates may be resolved as class II crossovers via partially redundant pathways involving MUS81 and FANCD2. This model is based on that described in (Mercier et al. 2014), incorporating data from (Morgan et al. 2021).

Meiosis begins with two key steps: firstly, each chromosome is tethered to a proteinaceous axis incorporating ASY1 (Armstrong et al. 2002) and ASY3 (Ferdous et al. 2012), and secondly, there is programmed induction of DNA double-strand breaks (DSBs) by a DNA topoisomerase VI–like complex comprising SPO11 (Keeney et al. 1997) and MTOPVIB (Vrielynck et al. 2016). Plants have three SPO11 homologs two of which, SPO11-1 and SPO11-2, form part of the meiotic topoisomerase complex and are required for meiotic double-strand break induction (Fig. 1; Grelon et al. 2001; Stacey et al. 2006), while the third, SPO11-3, has a non-meiotic role (Sugimoto-Shirasu et al. 2002). Following DSB induction, DNA ends are resected, and the single-stranded overhangs generated are bound by DMC1 and RAD51 which mediate inter-homologue strand-invasion forming a D-loop structure (Fig. 1; Shinohara et al. 1992; Ogawa et al. 1993; Sehorn et al. 2004; Da Ines et al. 2013). These joint molecules are then processed by numerous parallel pathways to be resolved as either crossovers or non-crossovers (Fig. 1; Bishop and Zickler 2004). While hundreds of DSBs are introduced at the beginning of meiosis, from ~ 200 in Arabidopsis (Kurzbauer et al. 2012; Girard et al. 2015) and ~ 400 in rice (Ren et al. 2021) to 750–1500 in wheat (Benyahya et al. 2020; Osman et al. 2021), only a very small percentage of these eventually become crossovers, with the balance of crossovers to non-crossovers determined by two antagonistic processes. On one hand, several helicases and associated proteins disassemble recombination intermediates and promote non-crossover formation via synthesis dependent strand annealing (SDSA) (Fig. 1; Crismani et al. 2012; Girard et al. 2015; Seguela-Arnaud et al. 2015; Fernandes et al. 2018b). In parallel, a subset of joint-molecules enters the major recombination pathway which in plants contributes around 70–90% of crossovers (Fig. 1; Mercier et al. 2005; Lhuissier et al. 2007; Falque et al. 2009). This pathway—referred to as the class I, or ZMM pathway—is defined by its reliance on a structurally diverse, yet functionally related group of proteins referred to collectively as the ZMMs (Sym et al. 1993; Ross-Macdonald and Roeder 1994; Hollingsworth et al. 1995; Chua and Roeder 1998; Nakagawa and Ogawa 1999; Agarwal and Roeder 2000; Tsubouchi et al. 2006; Lynn et al. 2007; De Muyt et al. 2018). In Arabidopsis, the ZMM proteins are SHOC1 (Macaisne et al. 2011), HEI10 (Chelysheva et al. 2012), ZIP4(SPO22) (Chelysheva et al. 2007), PTD (Macaisne et al. 2011), MER3 (Mercier et al. 2005), MSH4 (Higgins et al. 2004) and MSH5 (Higgins et al. 2008). The roles of these proteins are mostly conserved in yeast, animals and plants though there are some differences. For example, the transverse filament protein Zip1 (Sym et al. 1993) is absolutely required for class I crossover formation in yeast and considered a ZMM, while the Arabidopsis and rice homologs, ZYP1 and ZEP1, respectively, are dispensable class I crossover formation (Wang et al. 2010; Capilla-Pérez et al. 2021; France et al. 2021).

**Fig. 2:**
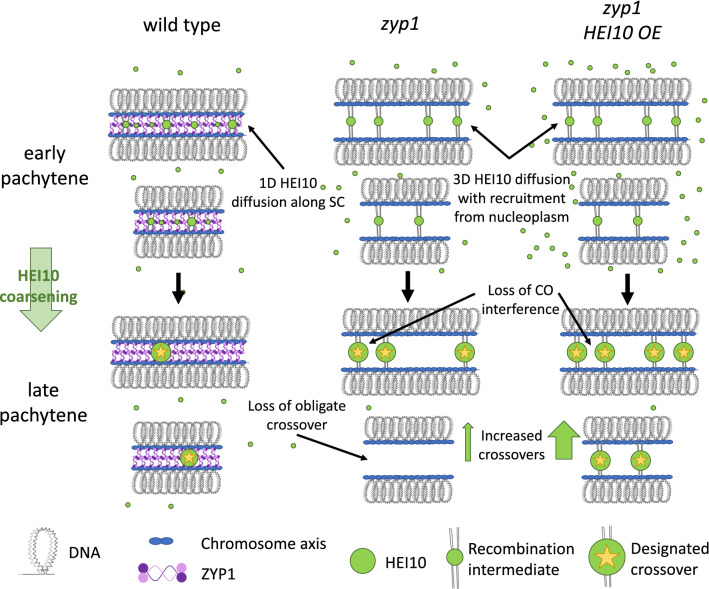
The HEI10 diffusion model of crossover patterning applied to meiosis in wild type,* zyp1* and* zyp1/HEI10* overexpression (OE) lines. Schematic representations of HEI10 localisation are based on cytological observations in wild type, *zyp1* and *zyp1/HEI10 OE* Arabidopsis. In all genotypes, HEI10 localises to recombination intermediates in early pachytene. In wild type, HEI10 is also distributed along the synaptonemal complex (SC). As pachytene progresses, there is “coarsening” of HEI10 foci with smaller foci being lost and larger foci increasing in size. By late pachytene, HEI10 only localises to sites of crossover (CO) designation. In wild type, restricting HEI10 diffusion to the single dimension of the SC ensures formation of the obligatory crossover, imposes crossover interference, and limits crossover number. In the absence of ZYP1, 3D diffusion of HEI10 with recruitment from the nucleoplasm results in loss of crossover interference and crossover assurance. In this context, crossover number is limited by HEI10 concentration, resulting in massively increased crossovers in *zyp1/HEI10* OE lines

A primary role of the ZMM proteins is to bind to and stabilise recombination intermediates formed between homologous chromosomes. In yeast, ZMM proteins also provide a functional link between meiotic crossover formation and assembly of the synaptonemal complex—the highly ordered and evolutionarily conserved structure which “zips” together homologous chromosomes in early meiotic prophase (Pyatnitskaya et al. 2022). This role of ZMMs in establishing a link between recombination intermediates and SC polymerisation might not be conserved in plants, however, as plant *zmm* mutants do not show defects in synapsis (Higgins et al. 2004, 2008; Chelysheva et al. 2007, 2012; Wang et al. 2012; Gonzalo et al. 2019; Desjardins et al. 2020). The majority of the ZMM proteins have been shown to directly bind to, or act on DNA recombination intermediates. This includes Mer3 which recognises and migrates D loops (Mazina et al. 2004), the Msh4-Msh5 heterodimer which stabilises double Holiday Junctions (dHJs) (Snowden et al. 2004) and Zip2(AtSHOC1) and Spo16(AtPTD) which bind both dHJs and D-loops (De Muyt et al. 2018). Zip4, a tetratricopeptide repeat containing protein, appears to play a scaffolding role in yeast, mediating interactions between recombination intermediates (via direct interactions with MSH5, ZIP2[AtSHOC1] and Spo16[AtPTD]), the chromosome axis (via direct interaction with Red1[AtASY3]) and the synaptonemal complex (via direct interactions with Emc11 and Gcm2) (De Muyt et al. 2018; Pyatnitskaya et al. 2022). Zip4 also directly interacts with the E3 ligase Zip3 which at late pachytene localises to sites of crossover formation (De Muyt et al. 2018). Through these multiple interactions, Zip4 links the stabilisation of recombination intermediates to crossover formation and assembly of the synaptonemal complex (Pyatnitskaya et al. 2022). HEI10, the plant functional orthologue of yeast Zip3, is a dosage dependent regulator of crossover formation (Chelysheva et al. 2012; Ziolkowski et al. 2017). It initially loads as many small foci on synapsed chromosomes but by late pachytene is found exclusively as large foci at sites of class I crossover formation (Fig. 1; Fig. 2; Chelysheva et al. 2012; Morgan et al. 2021). At this stage, HEI10 foci co-localise with MutL Homologue proteins MLH1 and MLH3, which also form very large foci at sites of class I crossover formation (Lhuissier et al. 2007; Chelysheva et al. 2010, 2012; Wang et al. 2012; Morgan et al. 2021; Osman et al. 2021). The size of these foci suggests the proteins are not purely playing enzymatic roles in resolving dHJs, and it is possible this size may be related to the exclusion of factors promoting non-crossover formation by SDSA or dHJ dissolution (Snowden et al. 2004). While the ZMM or class I recombination pathway accounts for the majority of crossovers in plants, about 10–30% of crossovers occur via a non-ZMM pathway (Mercier et al. 2005; Lhuissier et al. 2007; Falque et al. 2009). These crossovers are commonly referred to as class II crossovers or “non-interfering” crossovers as, unlike ZMM-dependent crossovers, they are not subject to crossover interference (see below). In Arabidopsis, these crossovers are dependent on partially redundant pathways involving MUS81 and FANCD2 (Kurzbauer et al. 2018).

### Crossover patterning

A striking feature of most plant recombination landscapes is their uneven distribution. This is particularly evident for plants with large genomes and extensive repeat-rich peri-centromeric regions. For these species, including many crops such as barley (The International Barley Genome Sequencing Consortium 2012), wheat (Appels et al. 2018), maize (Li et al. 2015), cotton (Shen et al. 2017) and tomato (The Tomato Genome Consortium 2012), there is a remarkably consistent pattern of recombination at the chromosomal scale, with high recombination rates in distal regions and large pericentromeric regions with little to no recombination. Gene density shows a similar pattern with gene density highest toward the telomeres and lowest toward the centromere. However, in most species, the rate of crossover formation declines more rapidly than gene density so that a considerable proportion of genes are located in low recombination regions. In barley and maize for example, around 20% of genes are in peri-centromeric regions which receive very few crossovers (The International Barley Genome Sequencing Consortium 2012; Bauer et al. 2013). This has considerable consequences particularly for plant breeding where the lack of recombination makes genes in these regions effectively inaccessible to breeders (Blary and Jenczewski 2019).

There are two main levels at which crossover patterning is influenced and these can be loosely separated into global factors and chromosomal feature-based factors. In recent years, new molecular, cytological and genomics approaches have greatly increased our understanding of the key players influencing both levels of crossover patterning.

### Global crossover patterning

At the global scale, crossover patterning is largely influenced by the classically observed phenomena of crossover interference, crossover homeostasis and the obligatory crossover and recent studies of plant meiosis have highlighted HEI10 and ZYP1 as key players in their implementation (Ziolkowski et al. 2017; Capilla-Pérez et al. 2021; France et al. 2021; Morgan et al. 2021). The following section provides an overview of these processes which between them determine the absolute number of crossovers, their distribution among chromosomes and inter-crossover distances, and discusses the implications of recent studies for our understanding of their molecular basis.

#### The obligatory crossover

Darlington and Dark (1932) made the early observation that crossovers are not randomly distributed among chromosomes, but that shorter chromosomes receive more crossovers per unit length and a minimum of at least one. They realised this was an important adaptation, as forming at least one crossover per bivalent ensures accurate segregation so that each chromosome remains “part of the permanent chromosome complement”. This idea was later coined the “obligatory crossover” (Owen 1949). An outcome of the obligatory crossover (also pointed out by Darlington and Dark) is that chromosomes have a minimum genetic map length of 50 cM (Darlington and Dark 1932).

#### Crossover homeostasis

In many species, variations in the number of meiotic DSBs (i.e. crossover precursors) have no appreciable impact on the number of crossovers (Martini et al. 2006; Rosu et al. 2011; Cole et al. 2012). This phenomenon is referred to as crossover homeostasis. In budding yeast, an 80% reduction in the number of meiotic DSBs results in only ~ 15% fewer crossovers (Martini et al. 2006), and in *Caenorhabditis elegans,* a single DSB is sufficient to ensure a crossover (Rosu et al. 2011). In plants, reports of crossover homeostasis vary somewhat, and interpretation is challenging due to the poor DSB quantification levels available. It has been reported to be a factor to at least some degree in Arabidopsis, where a 46% decrease in meiotic DSBs results in only a 17% reduction in class I crossovers (Xue et al. 2018). Similarly, a study of Arabidopsis *fas1* mutants, which have a ~ 40–50% increase in meiotic DSBs, showed no observable increase in chiasmata numbers (Varas et al. 2015). In maize though there is a correlation between DSB number and chiasmata number in different inbred lines, the increase in chiasmata is less than the increase in DSBs suggesting some homeostasis  (Sidhu et al. 2015). It seems likely therefore that crossover homeostasis is somewhat less strict in plants compared to other species, though still sufficient to ensure formation of the obligate crossover (Sidhu et al. 2015).

#### Crossover interference

In addition to being non-randomly distributed *among* chromosomes, crossovers are also non-randomly distributed *along* chromosomes. This is primarily due to the phenomenon of crossover interference, which was first reported by Sturtevant over a century ago in his studies of linkage in Drosophila (Sturtevant 1913). As well as producing the first ever genetic map, Sturtevant noted that the presence of a crossover in one genetic interval reduced the likelihood of a second crossover in an adjacent interval (Sturtevant 1913). This phenomenon, termed “interference” several years later (Muller 1916), results in crossover spacing that is more uniform than expected by chance.

The effect of interference on crossover patterning has been modelled using numerous approaches, two notable implementations being the gamma model and the beam-film model. The statistical gamma model is based on the observation that inter-crossover distances are relatively uniform, following a gamma distribution (Mcpeek and Speed 1995; Broman and Weber 2000; Housworth and Stahl 2003). In contrast, the beam-film model is a mechanistic model based on the redistribution of mechanical stress or crossover promoting force which is relieved locally following crossover designation (Kleckner et al. 2004; Zhang et al. 2014a). While these and other phenomenological and mechanistic models of interference have existed for some time (excellently (reviewed in Otto and Payseur 2019)), a biological understanding of interference is only just beginning to emerge.

A confounding factor for understanding the biological basis of crossover interference is the presence of both interfering and non-interfering crossovers. Anything that changes the ratio of interfering (class I) to non-interfering crossovers (class II) changes genetic readouts of crossover interference even if there has been no change in the underlying biological interference mechanisms. Clear examples of this are hyper-recombinant mutants which have increased levels of class II crossovers (Crismani et al. 2012; Girard et al. 2015; Seguela-Arnaud et al. 2015; Séguéla-Arnaud et al. 2017; Fernandes et al. 2018b). In these plants, class I crossover numbers remain largely unchanged and from a mechanistic standpoint crossover interference is operating essentially as normal. However, due to the large number of non-interfering crossovers, interference is entirely absent when measured genetically (Crismani et al. 2012; Séguéla-Arnaud et al. 2017; Fernandes et al. 2018b). There are several notable recent exceptions, however, which do illuminate mechanisms of crossover interference, and both implicate a role for the synaptonemal complex.

## Crossover interference and the synaptonemal complex

Within an organism, synaptonemal complex length is correlated with crossover number. This is true even when the same chromosome has a different synaptonemal complex length in different situations. For example, higher crossover rates are seen in budding yeast condensin mutants with longer synaptonemal complexes (Zhang et al. 2014b), and in barley, male meiosis has a longer synaptonemal complex and more crossovers at high temperature (Phillips et al. 2015). These observations have led to a model whereby crossover interference is imposed over a physical distance along chromosomes, i.e. µm chromosome axis or synaptonemal complex, rather than a genomic distance measured in Mb (Zickler and Kleckner 2015).

Differences in synaptonemal complex length are also associated with sex-specific differences in crossover rate, known as heterochiasmy. Sex-specific recombination rates have been described in both animals and plants (Singer et al. 2002; Tease and Hultén 2004; Lenormand and Dutheil 2005; Giraut et al. 2011; Tortereau et al. 2012; Gruhn et al. 2013; Phillips et al. 2015) and in the species where it has been investigated, including Arabidopsis, barley, mice and humans, the length of the synaptonemal complex is longer in the sex with more crossovers (Tease and Hultén 2004; Giraut et al. 2011; Gruhn et al. 2013; Phillips et al. 2015). In Arabidopsis, the difference in crossover rate between the sexes is particularly marked, with male meiosis having around 40% more crossovers than female meiosis (Giraut et al. 2011). In addition to differences in crossover number, there are also big differences in crossover distribution; crossover rates are highest in distal regions in Arabidopsis male meiosis, while in female meiosis crossover rates are highest adjacent to the peri-centromere (Giraut et al. 2011). Modelling of Arabidopsis meiosis has demonstrated that sex-specific differences in both crossover number and distribution can be entirely explained by differences in synaptonemal complex length if crossover interference is imposed over the same physical distance in micrometres (Giraut et al. 2011; Lloyd and Jenczewski 2019).

The relationship between genome size and the length of the chromosome axis and synaptonemal complex length is determined by the size and number of chromatin loops, which occur at an average density of around 20 loops per µm synaptonemal complex across a wide range of organisms (Zickler and Kleckner 1999). The larger the average size of the DNA loops, the shorter the eventual length of the synaptonemal complex for a given chromosome. While exact loop size and position likely vary from cell to cell (Schalbetter et al. 2019) and across meiotic stages (Zuo et al. 2021), sex-specific differences in loop size appear to be set very early in, or prior to, meiosis (Gruhn et al. 2013; Zickler and Kleckner 2015). This suggests that differences in crossover patterning attributable to differences in synaptonemal complex length, e.g. heterochiasmy (Drouaud et al. 2007; Giraut et al. 2011; Lloyd and Jenczewski 2019; Capilla-Pérez et al. 2021) or temperature induced changes (Phillips et al. 2015) may also be imposed very early in, or prior to, meiosis.

### ZYP1 and crossover interference

Our molecular understanding of how crossover interference might be mediated by the synaptonemal complex in plants was recently advanced with two papers characterising *zyp1* mutants in Arabidopsis (Capilla-Pérez et al. 2021; France et al. 2021). These studies show that in the absence of ZYP1, the transverse filament protein of the synaptonemal complex, the number of class I crossovers increase by around 50–70% and their positioning becomes random (Fig. 2), demonstrating that the synaptonemal complex is required for the imposition of crossover interference (Capilla-Pérez et al. 2021; France et al. 2021). Interestingly, in the absence of ZYP1, crossovers are not only randomly distributed along chromosomes but also between chromosomes, resulting in univalents at metaphase I even though overall crossover numbers are increased (Capilla-Pérez et al. 2021; France et al. 2021). This suggests that crossover interference and formation of the obligatory crossover are intimately linked, with ZYP1 required for both processes.

Another feature lost in *zyp1* mutants is heterochiasmy. Female crossover rates in *zyp1* mutants increase more than male crossover rates in *zyp1* mutants such that total crossover number and crossover distribution in the two sexes are equivalent (Capilla-Pérez et al. 2021). Recently, it has been reported that despite the loss of heterochiasmy differences in axis length between male and female meiosis are maintained in the absence of ZYP1 (Durand et al. 2022, bioRxiv). This probably reflects the fact that sex-specific differences in DNA loop size are established prior to synapsis (Gruhn et al. 2013; Zickler and Kleckner 2015). It also indicates that differences in axis/synaptonemal complex length are not a consequence different crossover rates. Rather, these findings demonstrate that differences in synaptonemal complex length, together with crossover interference, mediate heterochiasmy.

The observation that ZYP1 mediates crossover interference in Arabidopsis contrasts with previous suggestions that the chromosome axis rather than the synaptonemal complex was likely to impose crossover interference (Bishop and Zickler 2004). These suggestions were based primarily on data from yeast which show that the crossover non-crossover decision appears to be made very early at the leptotene/zygotene transition with SC nucleation only occurring at sites destined to become crossovers (Zickler and Kleckner 1998, 1999; Bishop and Zickler 2004; Zhang et al. 2014b). This model is supported by studies in yeast showing that even in the absence of ZIP1, synaptonemal complex nucleation sites still show interference (Fung et al. 2004). The results of Capilla-Pérez et al (2021) and France et al (2021) argue against this model in Arabidopsis and instead suggest that **a)** the synaptonemal complex is absolutely required for crossover interference and **b)** that a large part of the crossover non-crossover decision occurs independently of crossover interference. It will be interesting therefore to see whether the role of ZYP1 in mediating interference is plant specific, though establishing whether this is the case is likely to be challenging. Unlike plants (Wang et al. 2010; Capilla-Pérez et al. 2021; France et al. 2021), most other organisms require the transverse filament protein for crossover formation (Tung and Roeder 1998; Page and Scott Hawley 2001; Macqueen et al. 2002; De Vries et al. 2005) making it impossible to determine its role in crossover interference using simple gene knockouts. Libuda et al (2013), however, saw an increase in class I crossovers and a decrease in interference in *C. elegans* with a partial knockdown of SYP-1, providing at least some evidence that the role of the transverse filament protein in mediating interference may not be restricted to plants.

Although crossover interference is completely lost in Arabidopsis *zyp1* mutants, the increase in crossovers seen is not particularly large—around a 50–70% increase resulting in 15–20 crossovers per meiosis (Capilla-Pérez et al. 2021; France et al. 2021). This means that even when interference is absent, the vast majority of the ~ 200 meiotic DSBs induced at the beginning of meiosis are still repaired as non-crossovers. This begs the question what then, other than crossover interference, limits crossover number?

### HEI10 and crossover interference

Recent evidence indicates that the key factor limiting crossover numbers in the absence of crossover interference is the E3 ligase and ZMM protein HEI10 (Chelysheva et al. 2012; Ziolkowski et al. 2017; Durand et al. 2022 bioRxiv). HEI10 is part of a family of proteins with two main sub-groups: HEI10/CCNB1IP1 and Zip3/RNF212. Of these, plants only encode a HEI10 family member (Chelysheva et al. 2012), budding yeast only retain a Zip3 family member, while mammalian genomes encode both (Toby et al. 2003; Reynolds et al. 2013). In Arabidopsis, HEI10 is known to regulate crossover number in a dosage dependent manner with *HEI10* overexpression more than doubling crossover number and *HEI10* heterozygotes displaying reduced crossover formation (Ziolkowski et al. 2017). Natural variation in Arabidopsis crossover number is also largely explained by the different *HEI10* alleles present in each ecotype (Ziolkowski et al. 2017). While crossover rates are greatly increased by *HEI10* over expression, crossover distribution is less affected. F2 (Sex-averaged) *HEI10* overexpression crossover distributions are a similar shape to those of wild-type Arabidopsis with very low crossover numbers in centromeric/peri-centromeric regions and highest recombination rates in distal regions (Ziolkowski et al. 2017).

Cytological studies show that in zygotene, HEI10 initially loads throughout synapsed regions co-localising with the synaptonemal complex (Wang et al. 2012; Chelysheva et al. 2012). By pachytene, numerous distinct HEI10 foci are observable against a background of very small foci along the length of the bivalent (Fig. 2; Wang et al. 2012; Chelysheva et al. 2012; Morgan et al. 2021). As pachytene continues, HEI10 foci decrease in number and increase in size and by late pachytene only mark sites of crossover formation (Fig. 2), co-localising with MLH1 (Wang et al. 2012; Chelysheva et al. 2012; Morgan et al. 2021). Recently, researchers investigating both Arabidopsis (Morgan et al. 2021) and *C. elegans* (Zhang et al. 2018, 2021 bioRxiv) have proposed a mechanistic model for crossover patterning which explains this progressive reduction in HEI10 foci throughout pachytene, involving diffusion-mediated “coarsening” of HEI10 along the single dimension of a synapsed bivalent. In the mathematical model developed by Morgan et al (2021), local regions of high HEI10 density (HEI10 foci) are positioned at immobile recombination intermediate sites (i.e. SPO11-dependent inter-homologue joint-molecules) along a bivalent, with HEI10 uniformly distributed at lower concentration across the rest of the bivalent length. This initial state reflects cytological observations of HEI10 distribution in early pachytene (Morgan et al. 2021). HEI10 is then allowed to diffuse along the bivalent absorbing into and escaping from foci at recombination intermediates. The rate of escape is slower for larger foci, and as a result, large foci grow at the expense of small foci until a relatively steady state is achieved where only a few HEI10 foci remain (Morgan et al. 2021). This mathematical model is supported by experimental data from *C. elegans*, which shows that the HEI10 orthologue ZHP-3 primarily diffuses along the single dimension of the SC and diffuses at a slower rate from large foci (Zhang et al. 2021, bioRxiv).

An attractive aspect of this model is that it can explain four key features of crossover patterning:

1. *Crossover interference:* As the diffusion of HEI10 is constrained to a single dimension, larger foci tend to grow bigger at the expense of adjacent smaller foci. This inhibitory effect results in greater distance between final foci than expected if the same number of foci were randomly positioned along the bivalent.
2. *The obligate crossover:* Given sufficient initial loading of HEI10, each bivalent forms at least one focus. Sufficient HEI10 is ensured by its loading along the full length of the synaptonemal complex. HEI10 cannot “escape” the synapsed bivalent with diffusion being restricted to one dimension. In this way, synapsis sequesters sufficient HEI10 to the bivalent to ensure formation of at least one crossover.
3. *Crossover homeostasis:* Crossover formation is constrained by the amount of HEI10 initially loaded on to the bivalent. Given equivalent starting levels of HEI10 (e.g. representing equivalent cellular concentrations of HEI10), the number of recombination intermediates has limited effect on the final number of crossovers (Morgan et al. 2021).
4. *Synaptonemal complex length*—*crossover number correlations:* Differences in synaptonemal complex length alters the amount of HEI10 a given bivalent can sequester. The longer the synaptonemal complex, the more HEI10. This would result in bivalents with longer synaptonemal complexes having more crossovers.

## A HEI10 diffusion-based model of crossover patterning could account for many *zyp1* phenotypes

Another attractive feature of the HEI10 coarsening model is that it can explain the outcomes observed in the absence of ZYP1 and synapsis (Fozard et al. 2022, bioRxiv). When ZYP1 is absent, HEI10 is not found as small foci along the full length of the bivalent at early pachytene-like stages but instead localises as fewer large foci positioned between the two chromosome axes (Fig. 2), presumably at recombination intermediates (Capilla-Pérez et al. 2021). Despite loss of the synaptonemal complex in *zyp1* mutants, the HEI10 foci still increase in intensity as the pachytene-like stage progresses (Fig. 2; Capilla-Pérez et al. 2021) suggesting that HEI10 diffusion is still taking place but in three-dimensions rather than in one.

Three-dimensional diffusion of HEI10 throughout the nucleoplasm would allow HEI10 to move between foci on different chromosomes which would have several consequences. Firstly, it would prevent bivalents sequestering HEI10, resulting in crossovers being randomly distributed among bivalents. This would disrupt formation of the obligate crossover, resulting in **a)** univalents being observed at metaphase I and **b)** more univalents in *zyp1* mutants with a lower HEI10 dosage (e.g. more univalents in Ler-1 *zyp1* than Col-0 *zyp1*) both of which have been shown for *zyp1* mutants experimentally (Ziolkowski et al. 2017; Capilla-Pérez et al. 2021; France et al. 2021). Secondly, three-dimensional diffusion would enable HEI10 to migrate between any two foci in the cell, rather than just neighbouring foci. This would stop one HEI10 focus preferentially inhibiting the growth of an adjacent HEI10 focus and in doing so disrupt crossover interference (Fozard et al. 2022, bioRxiv), again, as shown experimentally for *zyp1* mutants (Capilla-Pérez et al. 2021; France et al. 2021). Thirdly, crossover formation on a given bivalent would not be limited by the amount of HEI10 that could be sequestered by synapsis, but rather cellular crossover levels would be limited by the total level of HEI10 in the nucleus. Assuming equal concentrations of HEI10 in male and female pachytene nuclei, this would abolish heterochiasmy; this abolition is also observed experimentally in *zyp1* mutants (Capilla-Pérez et al. 2021; France et al. 2021). For the same reason, the HEI10 diffusion model would predict no-change or a modest increase in crossover number in the absence of crossover interference (i.e. due to the limited pool of HEI10), such as is seen in *zyp1* mutants (Capilla-Pérez et al. 2021; France et al. 2021). These predictions contrast with those of the standard beam-film model which predicts a large increase in crossovers and maintenance of the obligate crossover in the absence of interference. These two models are not mutually exclusive however, for example it could be possible that HEI10 diffusion and/or coarsening are influenced by mechanical stress.

Another prediction of the HEI10 coarsening model is that overexpression of HEI10 in the absence of ZYP1 and synapsis should produce very large numbers of class I crossovers in both male and female meiosis. A recent pre-print reports this exact experiment in Arabidopsis (Durand et al. 2022, bioRxiv) observing an average of ~ 45 MLH1 foci in both sexes, representing a 6.7-fold in female and a 3.5-fold increase in male compared to their respective wild types. Clearly, the potential for the HEI10 coarsening-model to explain so many aspects of recombination patterning in both wild-type and mutant meiosis is encouraging. Further investigations will help to test the assumptions and predictions of the model.

### Feature-based crossover patterning

In addition to the global crossover patterning processes described above, genomic and epi-genomic features such as chromatin state, replication timing, gene density and many others are predictive of crossover patterning at both chromosomal and local scales (Choi et al. 2018; Underwood et al. 2018; Pratto et al. 2021; Hsu et al. 2022; Lian et al. 2022). In Arabidopsis, potato, wheat and maize crossovers have been shown to occur in open chromatin, at gene ends, i.e. transcription start sites and transcription termination sites, and in regions of lower than average DNA methylation (Choi et al. 2013; Yelina et al. 2015; Marand et al. 2017; Kianian et al. 2018; Tock et al. 2021). Identifying which factors directly influence local crossover patterning, however, is challenging, as so many genomic and epigenetic features show similar distribution patterns. For example, high gene density is associated with open chromatin, earlier replication timing, and euchromatic epigenetic marks and these are all negatively correlated with TE density, DNA methylation and heterochromatic histone marks. It is also likely that different genomic features influence different steps of the meiotic crossover pathway with, for example, a different set of genomic features influencing DSB formation and crossover designation. Despite these caveats, genomic features can be used to predict crossover patterning with considerable accuracy. In a recent report, authors used machine learning approaches to predict crossover distributions based on 17 genomic features (Lian et al. 2022). They determined the most informative features for predicting crossover distributions were open chromatin (ATAC-seq), gene density and CHH DNA methylation which together accounted for 85% of the variation in crossover distributions (Lian et al. 2022). These features have been shown experimentally to affect crossover patterning right from the beginning of the meiotic programme, shaping DSB formation at the sub-kilobase scale.

### Replication timing and open chromatin

In yeast, there is direct coupling between meiotic DNA replication and the initiation of recombination through DSB formation (Borde et al. 2000). This is triggered by phosphorylation of Mer2 in the wake of the replication fork which recruits the DSB machinery through a meiosis-specific and phosphorylation dependent direct interaction with Rec114 (Henderson et al. 2007; Murakami and Keeney 2014). It is unclear whether there is equivalent coupling between DNA replication and DSB induction in plants; however, if there is, there may well be some differences. Yeast interactions mediating the coupling between DNA replication and the initiation of recombination are not seen in yeast two hybrid experiments with the plant homologs, e.g. PRD3-PHS1, PRD3-PRD2 (Vrielynck et al. 2021), though it remains possible that (some of) these interactions do occur *in planta*. Recent genomics approaches (Repli-seq, Hansen et al. 2010) have enabled high-resolution elucidation of genome-wide replication timing and have shown that in mice and humans, crossover rates are highest in earlier replicating DNA (Pratto et al. 2021). At a broad scale, this is likely to be true in plants as well. Repli-seq studies of somatic cells in Arabidopsis and maize show that replication occurs earliest in transcriptionally active, gene and AT-rich open chromatin (Wear et al. 2017; Concia et al. 2018) showing strong similarities with crossover distributions (Kianian et al. 2018; Rowan et al. 2019). In both species, distal regions replicate earlier on average than interstitial and proximal regions (Wear et al. 2017; Concia et al. 2018) though the differences in replication rate between sub-telomeric and interstitial regions are much more marked in maize. While there have been no Repli-seq studies of *meiotic* DNA replication timing in plants, cytological studies (Higgins et al. 2012) suggest meiotic and somatic replication timing are likely to be similar.

### DSB patterning

In many mammals, the position of meiotic DSBs is determined by the zinc finger protein PRDM9 which recognises and binds a particular DNA sequence motif and then﻿ methylates nearby nucleosomes, altering local chromatin structure to enable access for SPO11 (Paigen and Petkov 2018). Plants do not have a PRDM9 homolog, and as a result, DSBs are not associated with a specific DNA sequence motif; however, chromatin accessibility is still a key determinant of DSB induction.

**Fig. 3:**
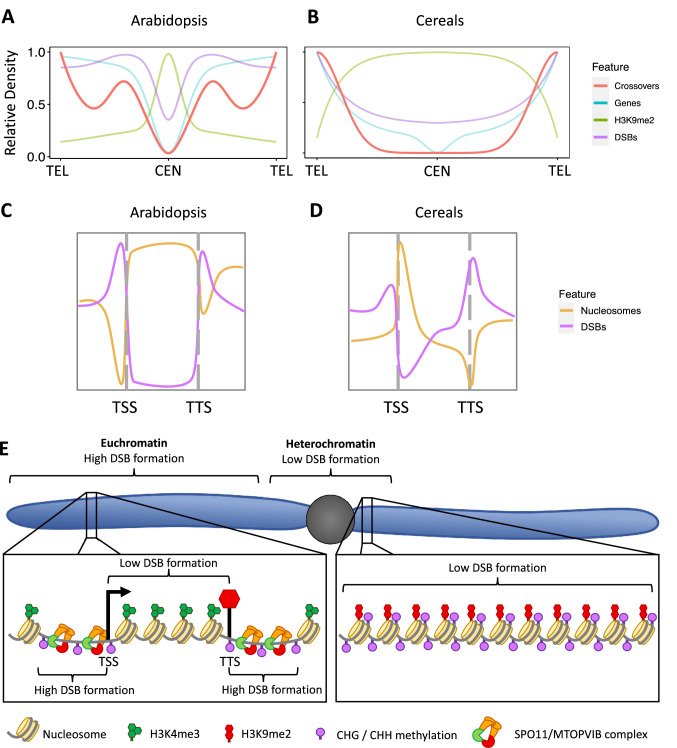
Genomic and epigenomic features influence plant crossover patterning. A-B: Representation of the relative densities of crossovers, genes, H3K9me2 and DSBs along a generic Arabidopsis (A) and cereal (B) chromosome; positions of telomeres (TEL) and Centromeres (CEN) are marked. C-D: Diagrammatic representation of local DSB and epigenomic feature density surrounding transcription start sites (TSS) and transcription termination sites (TTS) in Arabidopsis (C) and cereals (D). Contrasting DSB associations are observed at different scales (E). At the chromosomal scale, higher DSB levels are associated with epigenetic markers of euchromatin including low H3K9me2, high H3K4me3 and low non-CpG methylation. At the local (sub-kilobase) scale, DSBs in chromosome arms are associated with regions of low H3K4me3 and higher non-CpG methylation. At both chromosomal and local scales, DSBs are associated with regions of lower nucleosome occupancy

Spo11-oligo sequencing has been used to generate fine scale maps of DSB density in Arabidopsis. At the chromosome scale, DSB density is relatively constant, though it is reduced by around 40% in centromeric regions (Fig. 3; Choi et al. 2018). At the sub-kilobase scale, DSB density is far less uniform. In chromosome arms and pericentromeric regions, DSB density is enriched in gene promoters and terminators and is correlated with low nucleosome occupancy and low GC content (Fig. 3; Choi et al. 2018). While DSB density is associated with low nucleosome occupancy in all chromosomal regions, the picture is less straight forward for several common epigenetic markers. In pericentromeric and centromeric regions, both DSBs and crossovers are positively correlated with the euchromatic histone mark H3K4me3 and negatively correlated with markers of constitutive heterochromatin H3K9me2 and non-CG DNA methylation; however, these relationships are inverted in chromosome arms (Lambing et al. 2020a). The fact that DSBs and crossovers in Arabidopsis chromosome arms are negatively correlated with markers of euchromatin and positively correlated with markers of heterochromatin (Choi et al. 2018; Lambing et al. 2019) is puzzling given that globally, crossovers are associated with open chromatin. This is particularly true given that many analyses of Arabidopsis lines with altered chromatin state demonstrate that heterochromatin suppresses DSB formation (Yelina et al. 2015; Underwood et al. 2018). One epigenetic mark that is consistently (positively) correlated with DSB formation across the entire chromosome is H3K27me3 (Lambing et al. 2020a). Unlike markers of constitutive heterochromatin, H3K27me3, a mark of facultative heterochromatin, is enriched in chromosome arms (Zhang et al. 2007; Zheng and Chen 2011; Baker et al. 2015; Li et al. 2019).

The trends observed in Arabidopsis are likely to be broadly similar across most plants. DMC1 density in wheat, like Spo11-oligo density in Arabidopsis, is higher in gene promoters and terminators, regions with low nucleosome occupancy and high H3K27me3 (Fig. 3; Tock et al. 2021). While at the megabase scale DMC1 density is positively correlated with the euchromatic mark H3K4me3 and negatively correlated with CHG methylation, at the kilobase scale, DMC1 peak associations are inverted correlating with a local depletion of H3K4me3 and enrichment of CHG methylation (Tock et al. 2021). One explanation for these apparent contradictions and the similar contradictions observed in Arabidopsis relates to differences in how these epigenetic marks correlate with nucleosome occupancy at chromosomal and local scales (Fig. 3). At the chromosomal scale, high H3K4me3 signal indicates euchromatin and low nucleosome occupancy, while non-CG methylation indicates heterochromatin and high nucleosome occupancy (Fig. 3; Choi et al. 2018; Underwood et al. 2018; Tock et al. 2021). Contrastingly, at the kilobase scale, H3K4me3 (a component of nucleosomes) is absent from regions lacking nucleosomes and associates with regions of high nucleosome occupancy (Fig. 3) while non-CG methylation is enriched in plant promoters and terminators and is therefore associated with low nucleosome occupancy (Fig. 3; Lin et al. 2020; Lu et al. 2021). Thus, the apparent contradictions are all consistent with DSB formation occurring preferentially in open chromatin in gene promotors and terminators at sites of low nucleosome occupancy.

The positive association of H3K27me3 with both DMC1 and crossovers (and also Spo11-oligos in Arabidopsis) has led to the suggestion that in wheat, co-localisation of DSBs and H3K27me3-marked facultative heterochromatin promote crossover formation (Tock et al. 2021). In Arabidopsis, H3K27me3 is more uniformly distributed across chromosomes than in wheat where H3K27me3 shows a clear distal bias (Borg et al. 2020; Tock et al. 2021) and this reflects similar differences in their respective crossover landscapes (Rowan et al. 2019; Tock et al. 2021).

### Centromeric crossover suppression

In contrast to facultative heterochromatin, constitutive heterochromatin, highly enriched in plant centromeres and pericentromeres, is at least partly responsible for the suppression of crossovers (Henderson 2012; Fernandes et al. 2018a). For example, targeting RNA directed DNA methylation to euchromatic crossover hotspots in Arabidopsis converts these regions to constitutive heterochromatin (increased DNA methylation, H3K9me2 and increased nucleosome occupancy) resulting in reduced crossovers (Yelina et al. 2015). While DSBs are reduced in plant centromeres and the TE dense pericentromeric regions, the decrease (~ 40% in Arabidopsis) is relatively modest compared to the massive reduction in crossovers. This suggests that something else suppresses centromeric and pericentromeric crossovers in addition to DSB number. In Arabidopsis, mutants that lack H3K9me2 and non-CG methylation show both increased DSBs and crossovers in pericentromeres (Underwood et al. 2018; Lambing et al. 2020b) in contrast, loss of CG methylation (but not H3K9me2) in *met1* mutants results in increased DSBs but decreased crossovers (Underwood et al. 2018). It is possible therefore that H3K9me2 is the factor suppressing centromeric and pericentromeric crossovers. In this model, constitutive heterochromatin marks would suppress crossover designation independently of DSB formation.

### Sequence diversity

Another feature of plant genomes long known to be correlated with differences in crossover patterning is sequence diversity. A major example is inversions, which massively suppress crossovers in heterozygotes. A common example is seen in Arabidopsis recombination analyses using Col and Ler ecotypes which have a large inversion on the short arm of chromosome 4 (Giraut et al. 2011; Rowan et al. 2019). In many cases, crossovers do occur in inversions, but as they generate chromosomes lacking large numbers of genes and having duplicates of others, they do not result in viable gametes. The exception to this when inversions receive an even number of crossovers between the same two chromatids. This maintains the gene order of the two parental chromosomes and can therefore result in viable gametes (Termolino et al. 2019).

The other well-documented association is with SNP density, which in both Arabidopsis and wheat tends to be higher in regions with a higher crossover rate (Pont et al. 2019; Rowan et al. 2019; Blackwell et al. 2020; Tock et al. 2021). In some respects, this is puzzling as it has been well demonstrated that high levels of sequence divergence suppress meiotic recombination, for example low levels of recombination are observed in wheat-rye hybrids (Martín et al. 2014) and barley and tomato interspecific hybrids (Zhang et al. 1999; Tam et al. 2011). On the other hand, meiotic recombination is mutagenic (Arbeithuber et al. 2015) and thus may increase diversity in regions with higher crossover rates. A more detailed analysis shows that in Arabidopsis, crossover rates increase with SNP density from 0 to 0.5% but decrease with SNP densities above 0.5% (Blackwell et al. 2020). One possible unifying explanation is that SNPs accumulate in regions with more crossovers, with little effect on crossover rates until diversity reaches a critical threshold above which crossovers begin to be suppressed.

One counter argument to this explanation arises from the observation that in Arabidopsis *msh2* mutants, crossovers are reduced in SNP dense pericentromeric regions (Blackwell et al. 2020). It has been suggested therefore that moderate SNP density actively promotes crossover formation via an MSH2 dependent pathway (Blackwell et al. 2020). However, using EpiRILs (Colomé-Tatché et al. 2012) and more recently using EMS induced low density SNP markers (Lian et al. 2022), it has been shown that crossover rates are high near Arabidopsis pericentromeric regions even in the absence of SNPs, suggesting that higher SNP densities in these regions are a consequence rather than a cause of higher recombination rates. Interestingly, *ASY1*^+/-^ heterozygotes show a similar distal redistribution of crossovers to that seen in *msh2* mutants (Blackwell et al. 2020; Lambing et al. 2020a), suggesting reduced pericentromeric crossovers may be a common feature of minor perturbations to the meiotic programme in Arabidopsis. While most aspects of the correlation between SNP density and crossover rates can be explained by the mutagenic nature of crossovers, there remain gaps in our understanding. For example, a purely correlative relationship between SNP density and recombination rate does not account for the observation that crossovers are preferentially placed in regions of heterozygosity when they are juxtaposed with regions of homozygosity (Ziolkowski et al. 2015).

As most plant meiosis research is largely undertaken in selfing, highly inbred plants like Arabidopsis and cereal crops, it is easy to overlook the fact that levels of heterozygosity are considerably higher in predominantly outcrossing species, which constitute the majority (~ 70%) of plants (Goodwillie et al. 2005). For example, in the obligate out-crosser *A. arenosa,* coding sequences within a population diverge by around 1.5% at the nucleotide level (Monnahan et al. 2019) and presumably diverge by even more in non-coding sequences. It is unsurprising then that meiotic recombination has evolved to accommodate a relatively large degree of sequence divergence. Being too stringent in such species would result in segregation errors and loss of fertility due to loss of the obligate crossover (as is seen in inter-specific hybrids, e.g. Zhang et al. 1999). Although meiotic recombination can accommodate some heterozygosity, there are likely differences in how SNPs affect the patterning of crossovers occurring by the class I and class II recombination pathways. Arabidopsis *fancm, recq4* and *figl1* mutants have increased class II crossovers in inbred lines, but in intra-specific hybrids, they have no increase (*fancm*), or a smaller increase (*recq4*, *figl1*) (Fernandes et al. 2018b) suggesting that SNPs inhibit class II crossovers. As a result, class II crossovers are likely to occur at much lower frequency in outcrossing species with high heterozygosity. With their high stringency and (partial) dependence on MUS81 (Hartung et al. 2006; Kurzbauer et al. 2018), class II crossovers behave more like somatic recombination which is much more stringent than meiotic recombination. In Arabidopsis, even a single mismatch in a ~ 600 bp direct repeat is sufficient to reduce somatic recombination by 60–70% (Opperman et al. 2004).

### Interactions between global and feature based crossover patterning

While both global processes and chromosomal feature-based factors influence crossover patterning, these are not independent and likely interact at many levels. For example, the initial distribution of DSBs could influence the distribution of HEI10 loaded onto synapsed bivalents affecting downstream patterning processes. Another example relates to the spatiotemporal asymmetry in the progression of cereal meiosis which is likely initiated by differences in replication timing related to chromatin state. The spatial asymmetry of meiotic progression has been investigated in some detail in cereals where cytological studies have shown that the molecular events underlying meiotic recombination begin first in the sub-telomeric regions of each chromosome (Phillips et al. 2012; Osman et al. 2021). This is likely connected to the formation of the telomere bouquet, the characteristic clustering of telomeres in early meiotic prophase which is a universal feature of cereal meiosis, e.g. (Cowan and Zacheus Cande 2002; Phillips et al. 2010, 2012; Murphy and Bass 2012; Martin et al. 2017). Arabidopsis does form a telomere bouquet, but it its formation is less strict being observed in only around 50% of leptotene/zygotene nuclei and containing on average only around half of the telomeres (Hurel et al. 2018). In wheat and barley, the initial loading of the chromosome axis in G2, the induction of DSBs in leptotene, and synapsis in zygotene all begin from telomeric regions and progress toward the centromeres (Higgins et al. 2012; Osman et al. 2021). Distal regions are also the first to be replicated in cereals in both somatic (Wear et al. 2017) and pre-meiotic (Higgins et al. 2012) cells, suggesting that this asymmetry may be initiated pre-meiosis.

One outcome of the spatiotemporal asymmetry in synapsis is that HEI10 would load earlier (by ~ 4 h (Higgins et al. 2012)) in distal regions than proximal regions. Under the HEI10 coarsening model (Morgan et al. 2021), this could allow time for HEI10 diffusion-mediated coarsening in distal regions prior to proximal regions. It may also allow distal regions to sequester HEI10 in early zygotene, potentially reducing the availability of HEI10 to load in regions synapsing later in zygotene, mediating a HEI10 dosage gradient from telomeres to the centromere in early pachytene. Such a HEI10 concentration gradient is explicitly modelled in the HEI10 coarsening model and results in more distal crossovers when HEI10 concentration is higher at chromosome ends (Morgan et al. 2021). Earlier coarsening in distal regions (due to asynchronous synapsis and HEI10 loading) could similarly result in more distal crossovers. While detailed cytological analyses of HEI10 localisation and dynamics in cereals will be needed to validate this hypothesis experimentally, current evidence suggests HEI10 dynamics may largely mediate the formation of more crossovers in earlier synapsing regions in cereals.

## Conclusions

The patterning of meiotic crossovers is a highly regulated process and has important implications for patterns of inheritance and evolutionary outcomes. Crossover patterning is influenced both by globally acting processes, and genomic and epigenomic features which interact to set final crossover positions along chromosomes. Global crossover patterning is shaped by the well-described phenomena of crossover interference, the obligate crossover, crossover homeostasis and heterochiasmy, and while their basis has long been an enigma, recent studies have implicated ZYP1 and HEI10 as key players in their implementation. Crossover interference, the obligate crossover and heterochiasmy are all outcomes of the HEI10 coarsening model proposed by Morgan et al. (2021) and have all been shown experimentally to be mediated by ZYP1 (Capilla-Pérez et al. 2021; France et al. 2021). The potential for an extended HEI10 coarsening model to explain all phenotypes observed in *zyp1* mutants lends further weight to this description of crossover patterning and an important next step will be experiments testing the assumptions of the model in plants. Given the loss of the obligate crossover in *zyp1* mutants and the dosage dependent pro-crossover activity of HEI10, it is tempting to speculate that a key role of synapsis is to sequester sufficient HEI10 to each bivalent prior to crossover designation, to ensure all chromosomes receive at least one crossover, i.e. crossover assurance.

In addition to these global actors, crossover patterning at both chromosomal and local scales is shaped by numerous genomic and epigenomic features. Crossovers preferentially occur in open chromatin, in gene promoters and terminators and regions of low nucleosome occupancy. Although crossovers are associated with regions of higher SNP density, this is most likely non-causal and instead probably reflects the mutagenic nature of crossovers. However, there is still more to discover in this relationship as SNP density does appear to actively promote crossover formation in some contexts (Ziolkowski et al. 2015). One big remaining uncertainty is the identity of the factor suppressing centromeric and pericentromeric crossovers. There is good evidence that H3K9me2 may be key (Underwood et al. 2018), though it is also possible that a lack of crossover promotion (rather than suppression) in centromeres and pericentromeres (e.g. via H3K23me3) is behind their low crossover rate. Genome-wide studies of crossover formation in plants lacking H3K9me2 will go some way toward answering these questions.

## References

[CR1] Agarwal S, Roeder GS (2000). Zip3 provides a link between recombination enzymes and synaptonemal complex proteins. Cell.

[CR2] Appels R, Eversole K, Feuillet C (2018). Shifting the limits in wheat research and breeding using a fully annotated reference genome. Science.

[CR3] Arbeithuber B, Betancourt AJ, Ebner T, Tiemann-Boege I (2015). Crossovers are associated with mutation and biased gene conversion at recombination hotspots. Proc Natl Acad Sci.

[CR4] Armstrong SJ, Caryl AP, Jones GH, Franklin FCH (2002). Asy1, a protein required for meiotic chromosome synapsis, localizes to axis-associated chromatin in Arabidopsis and Brassica. J Cell Sci.

[CR5] Baker K, Dhillon T, Colas I (2015). Chromatin state analysis of the barley epigenome reveals a higher-order structure defined by H3K27me1 and H3K27me3 abundance. Plant J.

[CR6] Bauer E, Falque M, Walter H (2013). Intraspecific variation of recombination rate in maize. Genome Biol.

[CR7] Benyahya F, Nadaud I, Da Ines O (2020). SPO11.2 is essential for programmed double-strand break formation during meiosis in bread wheat (Triticum aestivum L.). Plant J.

[CR8] Bishop DK, Zickler D (2004). Early decision: Meiotic crossover interference prior to stable strand exchange and synapsis. Cell.

[CR9] Blackwell AR, Dluzewska J, Szymanska-Lejman M (2020). MSH2 shapes the meiotic crossover landscape in relation to interhomolog polymorphism in Arabidopsis. EMBO J.

[CR10] Blary A, Jenczewski E (2019). Manipulation of crossover frequency and distribution for plant breeding. Theor Appl Genet.

[CR11] Borde V, Goldman ASH, Lichten M (2000). Direct coupling between meiotic DNA replication and recombination initiation. Science.

[CR12] Borg M, Jacob Y, Susaki D (2020). Targeted reprogramming of H3K27me3 resets epigenetic memory in plant paternal chromatin. Nat Cell Biol.

[CR13] Broman KW, Weber JL (2000). Characterization of Human Crossover Interference. Am J Hum Genet.

[CR14] Capilla-Pérez L, Durand S, Hurel A (2021). The synaptonemal complex imposes crossover interference and heterochiasmy in Arabidopsis. Proc Natl Acad Sci U S A.

[CR15] Chelysheva L, Gendrot G, Vezon D (2007). Zip4/Spo22 is required for class I CO formation but not for synapsis completion in Arabidopsis thaliana. PLoS Genet.

[CR16] Chelysheva L, Grandont L, Vrielynck N (2010). An easy protocol for studying chromatin and recombination protein dynamics during Arabidopsisthaliana meiosis: Immunodetection of cohesins, histones and MLH1. Cytogenet Genome Res.

[CR17] Chelysheva L, Vezon D, Chambon A (2012). The Arabidopsis HEI10 is a new ZMM protein related to Zip3. PLoS Genet.

[CR18] Choi K, Zhao X, Kelly KA (2013). Arabidopsis meiotic crossover hot spots overlap with H2A.Z nucleosomes at gene promoters. Nat Genet.

[CR19] Choi K, Zhao X, Tock AJ (2018). Nucleosomes and DNA methylation shape meiotic DSB frequency in Arabidopsis thaliana transposons and gene regulatory regions. Genome Res.

[CR20] Chua PR, Roeder GS (1998). Zip2, a Meiosis-Specific Protein Required for the Initiation of Chromosome Synapsis. Cell.

[CR21] Cole F, Kauppi L, Lange J (2012). Homeostatic control of recombination is implemented progressively in mouse meiosis. Nat Cell Biol.

[CR22] Colomé-Tatché M, Cortijo S, Wardenaar R (2012). Features of the Arabidopsis recombination landscape resulting from the combined loss of sequence variation and DNA methylation. Proc Natl Acad Sci U S A.

[CR23] Concia L, Brooks AM, Wheeler E (2018). Genome-wide analysis of the arabidopsis replication timing program. Plant Physiol.

[CR24] Cowan CR, Zacheus Cande Z (2002). Meiotic telomere clustering is inhibited by colchicine but does not require cytoplasmic microtubules. J Cell Sci.

[CR25] Crismani W, Girard C, Froger N (2012). FANCM limits meiotic crossovers. Science.

[CR26] Da Ines O, Degroote F, Goubely C (2013). Meiotic recombination in arabidopsis is catalysed by DMC1, with RAD51 Playing a Supporting Role. PLoS Genet.

[CR27] Darlington CD, Dark SO (1932). the Origin and Behaviour of Chiasmata II. Stenobothrus Parallelus. Cytologia (tokyo).

[CR28] De Muyt A, Pyatnitskaya A, Andréani J (2018). A meiotic XPF–ERCC1-like complex recognizes joint molecule recombination intermediates to promote crossover formation. Genes Dev.

[CR29] De Vries FAT, De Boer E, Van Den Bosch M (2005). Mouse Sycp1 functions in synaptonemal complex assembly, meiotic recombination, and XY body formation. Genes Dev.

[CR30] Desjardins SD, Ogle DE, Ayoub MA (2020). MutS homologue 4 and MutS homologue 5 maintain the obligate crossover in wheat despite stepwise gene loss following polyploidization. Plant Physiol.

[CR31] Drouaud J, Mercier R, Chelysheva L (2007). Sex-specific crossover distributions and variations in interference level along Arabidopsis thaliana chromosome 4. PLoS Genet.

[CR32] Durand S, Lian Q, Jing J, et al (2022) Dual control of meiotic crossover patterning. bioRxiv 10.1038/s41467-022-33472-w PMC9556546 36224180

[CR33] Falque M, Anderson LK, Stack SM (2009). Two Types of Meiotic Crossovers Coexist in Maize. Plant Cell.

[CR34] Ferdous M, Higgins JD, Osman K (2012). Inter-homolog crossing-over and synapsis in Arabidopsis meiosis are dependent on the chromosome axis protein AtASY3. PLoS Genet.

[CR35] Fernandes JB, Duhamel M, Seguéla-Arnaud M (2018). FIGL1 and its novel partner FLIP form a conserved complex that regulates homologous recombination. PLoS Genet.

[CR36] Fernandes JB, Seguéla-Arnaud M, Larchevêque C (2018). Unleashing meiotic crossovers in hybrid plants. Proc Natl Acad Sci.

[CR37] Fozard JA, Morgan C, Howard M (2022) The synaptonemal complex controls cis-versus trans- interference in coarsening-based meiotic crossover patterning. bioRxiv. 10.1101/2022.04.11.487855

[CR38] France MG, Enderle J, Röhrig S (2021). ZYP1 is required for obligate cross-over formation and cross-over interference in Arabidopsis. Proc Natl Acad Sci U S A.

[CR39] Fung JC, Rockmill B, Odell M, Roeder GS (2004). Imposition of crossover interference through the nonrandom distribution of synapsis initiation complexes. Cell.

[CR40] Girard C, Chelysheva L, Choinard S (2015). AAA-ATPase FIDGETIN-LIKE 1 and Helicase FANCM Antagonize Meiotic Crossovers by Distinct Mechanisms. PLOS Genet.

[CR41] Giraut L, Falque M, Drouaud J (2011). Genome-wide crossover distribution in Arabidopsis thaliana meiosis reveals sex-specific patterns along chromosomes. PLoS Genet.

[CR42] Gonzalo A, Lucas M, Charpentier C (2019). Reducing MSH4 copy number prevents meiotic crossovers between non-homologous chromosomes in Brassica napus. Nat Commun.

[CR43] Goodwillie C, Kalisz S, Eckert CG (2005). The evolutionary enigma of mixed mating systems in plants: Occurrence, theoretical explanations, and empirical evidence. Annu Rev Ecol Evol Syst.

[CR44] Grelon M, Vezon D, Gendrot G, Pelletier G (2001). AtSPO11-1 is necessary for efficient meiotic recombination in plants. EMBO J.

[CR45] Gruhn JR, Rubio C, Broman KW (2013). Cytological studies of human meiosis: Sex-specific differences in recombination originate at, or prior to, establishment of double-strand breaks. PLoS ONE.

[CR46] Hansen RS, Thomas S, Sandstrom R (2010). Sequencing newly replicated DNA reveals widespread plasticity in human replication timing. Proc Natl Acad Sci U S A.

[CR47] Hartung F, Suer S, Bergmann T, Puchta H (2006). The role of AtMUS81 in DNA repair and its genetic interaction with the helicase AtRecQ4A. Nucleic Acids Res.

[CR48] Henderson IR (2012). Control of meiotic recombination frequency in plant genomes. Curr Opin Plant Biol.

[CR49] Henderson KA, Kee K, Maleki S (2007). Cyclin-dependent kinase directly regulates initiation of meiotic recombination. Cell.

[CR50] Higgins JD, Armstrong SJ, Franklin FCH, Jones GH (2004). The Arabidopsis MutS homolog AtMSH4 functions at an early step in recombination: Evidence for two classes of recombination in Arabidopsis. Genes Dev.

[CR51] Higgins JD, Perry RM, Barakate A (2012). Spatiotemporal Asymmetry of the Meiotic Program Underlies the Predominantly Distal Distribution of Meiotic Crossovers in Barley. Plant Cell.

[CR52] Higgins JD, Vignard J, Mercier R (2008). AtMSH5 partners AtMSH4 in the class I meiotic crossover pathway in Arabidopsis thaliana, but is not required for synapsis. Plant J.

[CR53] Hollingsworth NM, Ponte L, Halsey C (1995). MSH5, a novel MutS homolog, facilitates meiotic reciprocal recombination between homologs in Saccharomyces cerevisiae but not mismatch repair. Genes Dev.

[CR54] Housworth EA, Stahl FW (2003). Crossover Interference in Humans. Am J Hum Genet.

[CR55] Hsu Y-M, Falque M, Martin OC (2022). quantitative modelling of fine-scale variations in the arabidopsis thaliana crossover landscape. Quant Plant Biol.

[CR56] Hurel A, Phillips D, Vrielynck N (2018). A cytological approach to studying meiotic recombination and chromosome dynamics in Arabidopsis thaliana male meiocytes in three dimensions. Plant J.

[CR57] International wheat genome sequencing consortium (IWGSC) TIWGSC, Lobell DB, Schlenker W, et al (2014) A chromosome-based draft sequence of the hexaploid bread wheat Triticum aestivum genome. Science. 10.1126/science.1251788 10.1126/science.1251788 25035500

[CR58] Keeney S, Giroux CN, Kleckner N (1997). Meiosis-specific DNA double-strand breaks are catalyzed by Spo11, a member of a widely conserved protein family. Cell.

[CR59] Kianian PMA, Wang M, Simons K (2018). High-resolution crossover mapping reveals similarities and differences of male and female recombination in maize. Nat Commun.

[CR60] Kleckner N, Zickler D, Jones GH (2004). A mechanical basis for chromosome function. Proc Natl Acad Sci.

[CR61] Kurzbauer M-T, Pradillo M, Kerzendorfer C (2018). Arabidopsis thaliana FANCD2 Promotes Meiotic Crossover Formation. Plant Cell.

[CR62] Kurzbauer MT, Uanschou C, Chen D, Schlögelhofer P (2012). The recombinases DMC1 and RAD51 are functionally and spatially separated during meiosis in Arabidopsis. Plant Cell.

[CR63] Lambing C, Tock AJ, Choi K, et al (2019) REC8-cohesin , chromatin and transcription orchestrate meiotic recombination in the Arabidopsis genome 10.1105/tpc.19.00866 PMC7145502 32024691

[CR64] Lambing C, Kuo PC, Tock AJ (2020). ASY1 acts as a dosage-dependent antagonist of telomere-led recombination and mediates crossover interference in Arabidopsis. Proc Natl Acad Sci U S A.

[CR65] Lambing C, Tock AJ, Topp SD (2020). Interacting Genomic Landscapes of REC8-Cohesin, Chromatin, and Meiotic Recombination in Arabidopsis. Plant Cell.

[CR66] Lenormand T, Dutheil J (2005). Recombination difference between sexes: A role for haploid selection. PLoS Biol.

[CR67] Lhuissier FGP, Offenberg HH, Wittich PE (2007). The Mismatch Repair Protein MLH1 Marks a Subset of Strongly Interfering Crossovers in Tomato. Plant Cell Online.

[CR68] Li X, Li L, Yan J (2015). Dissecting meiotic recombination based on tetrad analysis by single-microspore sequencing in maize. Nat Commun.

[CR69] Li Z, Wang M, Lin K (2019). The bread wheat epigenomic map reveals distinct chromatin architectural and evolutionary features of functional genetic elements. Genome Biol.

[CR70] Lian Q, Solier V, Walkemeier B et al (2022) The megabase-scale crossover landscape is largely independent of sequence divergence. Nat Commun 13. 10.1038/s41467-022-31509-8 10.1038/s41467-022-31509-8 PMC9250513 35780220

[CR71] Libuda DE, Uzawa S, Meyer BJ, Villeneuve AM (2013). Meiotic chromosome structures constrain and respond to designation of crossover sites. Nature.

[CR72] Lin W, Sun L, Huang RZ (2020). Active DNA demethylation regulates tracheary element differentiation in Arabidopsis. Sci Adv.

[CR73] Lloyd AH, Jenczewski E (2019). Modelling sex-specific crossover patterning in Arabidopsis. Genetics.

[CR74] Lu FH, McKenzie N, Gardiner LJ (2021). Reduced chromatin accessibility underlies gene expression differences in homologous chromosome arms of diploid Aegilops tauschii and hexaploid wheat. Gigascience.

[CR75] Lynn A, Soucek R, Börner GV (2007). ZMM proteins during meiosis : Crossover artists at work. Chromosom Res.

[CR76] Macaisne N, Vignard J, Mercier R (2011). SHOC1 and PTD form an XPF – ERCC1-like complex that is required for formation of class I. Crossovers.

[CR77] Macqueen AJ, Colaiácovo MP, McDonald K, Villeneuve AM (2002). Synapsis-dependent and -independent mechanisms stabilize homolog pairing during meiotic prophase in C. Elegans.

[CR78] Marand AP, Jansky SH, Zhao H (2017). Meiotic crossovers are associated with open chromatin and enriched with Stowaway transposons in potato. Genome Biol.

[CR79] Martin AC, Rey MD, Shaw P, Moore G (2017). Dual effect of the wheat Ph1 locus on chromosome synapsis and crossover. Chromosoma.

[CR80] Martín AC, Shaw P, Phillips D (2014). Licensing MLH1 sites for crossover during meiosis. Nat Commun.

[CR81] Martini E, Diaz RL, Hunter N, Keeney S (2006). Crossover homeostasis in yeast meiosis. Cell.

[CR82] Mazina OM, Mazin AV, Nakagawa T (2004). Saccharomyces cerevisiae Mer3 helicase stimulates 3′-5′ heteroduplex extension by Rad 51: Implications for crossover control in meiotic recombination. Cell.

[CR83] McDonald MJ, Rice DP, Desai MM (2016). Sex speeds adaptation by altering the dynamics of molecular evolution. Nature.

[CR84] Mcpeek MS, Speed TP (1995). Modeling interference in genetic recombination. Genetics.

[CR85] Mercier R, Jolivet S, Vezon D (2005). Two meiotic crossover classes cohabit in arabidopsis : one is dependent on MER3, whereas the other one is not. Curr Biol.

[CR86] Mercier R, Mezard C, Jenczewski E (2014). The Molecular Biology of Meiosis in Plants. Annu Rev Plant Biol.

[CR87] Monnahan P, Kolář F, Baduel P (2019). Pervasive population genomic consequences of genome duplication in Arabidopsis arenosa. Nat Ecol Evol.

[CR88] Morgan C, Fozard JA, Hartley M (2021). Diffusion-mediated HEI10 coarsening can explain meiotic crossover positioning in Arabidopsis. Nat Commun.

[CR89] Muller HJ (1916). The Mechanism of Crossing-Over. II Am Nat.

[CR90] Murakami H, Keeney S (2014). Temporospatial coordination of meiotic dna replication and recombination via DDK recruitment to replisomes. Cell.

[CR91] Murphy SP, Bass HW (2012). The maize (Zea mays) desynaptic (dy) mutation defines a pathway for meiotic chromosome segregation, linking nuclear morphology, telomere distribution and synapsis. J Cell Sci.

[CR92] Nakagawa T, Ogawa H (1999). The Saccharomyces cerevisiae MER3 gene, encoding a novel helicase-like protein, is required for crossover control in meiosis. EMBO J.

[CR93] Ogawa T, Yu X, Shinohara A, Egelman EH (1993). Similarity of the yeast RAD51 filament to the bacterial RecA filament. Science.

[CR94] Opperman R, Emmanuel E, Levy AA (2004). The effect of sequence divergence on recombination between direct repeats in arabidopsis. Genetics.

[CR95] Osman K, Algopishi U, Higgins JD (2021). Distal Bias of meiotic crossovers in hexaploid bread wheat reflects spatio-temporal asymmetry of the meiotic program. Front Plant Sci.

[CR96] Otto SP, Payseur BA (2019). Crossover interference: shedding light on the evolution of recombination. Annu Rev Genet.

[CR97] Owen ARG (1949). A possible interpretation of the apparent interference across the centromere found by callan and montalenti in culex pipiens. Heredity (edinb).

[CR98] Page SL, Scott Hawley R (2001). c(3)G encodes a drosophila synaptonemal complex protein. Genes Dev.

[CR99] Paigen K, Petkov PM (2018). PRDM9 and Its role in genetic recombination. Trends Genet.

[CR100] Phillips D, Jenkins G, Macaulay M (2015). The effect of temperature on the male and female recombination landscape of barley. New Phytol.

[CR101] Phillips D, Nibau C, Ramsay L (2010). Development of a molecular cytogenetic recombination assay for barley. Cytogenet Genome Res.

[CR102] Phillips D, Nibau C, Wnetrzak J, Jenkins G (2012). High resolution analysis of meiotic chromosome structure and behaviour in barley (Hordeum vulgare L.). PLoS ONE.

[CR103] Pont C, Leroy T, Seidel M (2019). Tracing the ancestry of modern bread wheats. Nat Genet.

[CR104] Pratto F, Brick K, Cheng G (2021). Meiotic recombination mirrors patterns of germline replication in mice and humans. Cell.

[CR105] Pyatnitskaya A, Andreani J, Guérois R (2022). The Zip4 protein directly couples meiotic crossover formation to synaptonemal complex assembly. Genes Dev.

[CR106] Ren L, Zhao T, Zhao Y (2021). The E3 ubiquitin ligase DESYNAPSIS1 regulates synapsis and recombination in rice meiosis. Cell Rep.

[CR107] Reynolds A, Qiao H, Yang Y (2013). RNF212 is a dosage-sensitive regulator of crossing-over during mammalian meiosis. Nat Genet.

[CR108] Ross-Macdonald P, Roeder GS (1994). Mutation of a meiosis-specific MutS homolog decreases crossing over but not mismatch correction. Cell.

[CR109] Rosu S, Libuda DE, Villeneuve AM (2011). Robust crossover assurance and regulated interhomolog access maintain meiotic crossover number. Science.

[CR110] Rowan BA, Heavens D, Feuerborn TR (2019). An ultra high-density arabidopsis thaliana crossover. Genetics.

[CR111] Schalbetter SA, Fudenberg G, Baxter J (2019). Principles of meiotic chromosome assembly revealed in S. cerevisiae. Nat Commun.

[CR112] Séguéla-Arnaud M, Choinard S, Larchevêque C (2017). RMI1 and TOP3α limit meiotic CO formation through their C-terminal domains. Nucleic Acids Res.

[CR113] Seguela-Arnaud M, Crismani W, Mazel J (2015). Multiple mechanisms limit meiotic crossovers : TOP3 α and two BLM homologs antagonize crossovers in parallel to FANCM. Proc Natl Acad Sci.

[CR114] Sehorn MG, Sigurdsson S, Bussen W (2004). Human meiotic recombinase Dmc1 promotes ATP-dependent homologous DNA strand exchange. Nature.

[CR115] Shen C, Li X, Zhang R, Lin Z (2017). Genome-wide recombination rate variation in a recombination map of cotton. PLoS ONE.

[CR116] Shinohara A, Ogawa H, Ogawa T (1992). Rad51 protein involved in repair and recombination in S. cerevisiae is a RecA-like protein. Cell.

[CR117] Sidhu GK, Fang C, Olson MA (2015). Recombination patterns in maize reveal limits to crossover homeostasis. Proc Natl Acad Sci.

[CR118] Singer A, Perlman H, Yan Y (2002). Sex-specific recombination rates in zebrafish (Danio rerio). Genetics.

[CR119] Snowden T, Acharya S, Butz C (2004). hMSH4-hMSH5 recognizes holliday junctions and forms a meiosis-specific sliding clamp that embraces homologous chromosomes. Mol Cell.

[CR120] Stacey NJ, Kuromori T, Azumi Y (2006). Arabidopsis SPO11-2 functions with SPO11-1 in meiotic recombination. Plant J.

[CR121] Sturtevant AH (1913). The linear arrangement of six sex-linked factors in Drosophila, as shown by their mode of association. J Exp Zool.

[CR122] Sugimoto-Shirasu K, Stacey NJ, Corsar J (2002). DNA topoisomerase VI is essential for endoreduplication in Arabidopsis. Curr Biol.

[CR123] Sym M, Engebrecht J, Roeder GS (1993). ZIP1 is a synaptonemal complex protein required for meiotic chromosome synapsis. Cell.

[CR124] Tam SM, Hays JB, Chetelat RT (2011). Effects of suppressing the DNA mismatch repair system on homeologous recombination in tomato. Theor Appl Genet.

[CR125] Tease C, Hultén MA (2004). Inter-sex variation in synaptonemal complex lengths largely determine the different recombination rates in male and female germ cells. Cytogenet Genome Res.

[CR126] Termolino P, Falque M, Aiese Cigliano R (2019). Recombination suppression in heterozygotes for a pericentric inversion induces the interchromosomal effect on crossovers in Arabidopsis. Plant J.

[CR127] The International Barley Genome Sequencing Consortium (2012) A physical , genetic and functional sequence assembly of the barley genome. Nature 491:711–716. 10.1038/nature11543 10.1038/nature11543 23075845

[CR128] The Tomato Genome Consortium (2012). The tomato genome sequence provides insights into fleshy fruit evolution. Nature.

[CR129] Toby GG, Gherraby W, Coleman TR, Golemis EA (2003). A novel ring finger protein, human enhancer of invasion 10, alters mitotic progression through regulation of cyclin B levels. Mol Cell Biol.

[CR130] Tock AJ, Holland DM, Jiang W (2021). Crossover-active regions of the wheat genome are distinguished by DMC1, the chromosome axis, H3K27me3, and signatures of adaptation. Genome Res.

[CR131] Tortereau F, Servin B, Frantz L (2012). A high density recombination map of the pig reveals a correlation between sex-specific recombination and GC content. BMC Genomics.

[CR132] Tsubouchi T, Zhao H, Roeder GS (2006). The meiosis-specific zip4 protein regulates crossover distribution by promoting synaptonemal complex formation together with zip2. Dev Cell.

[CR133] Tung KS, Roeder GS (1998). Meiotic chromosome morphology and behavior in zip1 mutants of Saccharomyces cerevisiae. Genetics.

[CR134] Underwood CJ, Choi K, Lambing C (2018). Epigenetic activation of meiotic recombination near Arabidopsis thaliana centromeres via loss of H3K9me2 and non-CG DNA methylation. Genome Res.

[CR135] Varas J, Sánchez-Morán E, Copenhaver GP (2015). Analysis of the relationships between DNA double-strand breaks, synaptonemal complex and crossovers using the atfas1-4 mutant. PLoS Genet.

[CR136] Vrielynck N, Chambon A, Vezon D (2016). A DNA topoisomerase VI-like complex initiates meiotic recombination. Science.

[CR137] Vrielynck N, Schneider K, Rodriguez M (2021). Conservation and divergence of meiotic DNA double strand break forming mechanisms in arabidopsis thaliana. Nucleic Acids Res.

[CR138] Wang K, Wang M, Tang D (2012). The role of rice HEI10 in the formation of meiotic crossovers. PLoS Genet.

[CR139] Wang M, Wang K, Tang D (2010). The central element protein ZEP1 of the synaptonemal complex regulates the number of crossovers during meiosis in rice. Plant Cell.

[CR140] Wear EE, Song J, Zynda GJ (2017). Genomic analysis of the DNA replication timing program during mitotic S phase in maize (Zea mays) root tips. Plant Cell.

[CR141] Xue M, Wang J, Jiang L (2018). The number of meiotic double-strand breaks influences crossover distribution in arabidopsis. Plant Cell.

[CR142] Yelina NE, Lambing C, Hardcastle TJ (2015). DNA methylation epigenetically silences crossover hot spots and controls chromosomal domains of meiotic recombination in arabidopsis. Genes Dev.

[CR143] Zhang L, Stauffer W, Zwicker D, Dernburg AF (2021) Crossover patterning through kinase-regulated condensation and coarsening of recombination nodules. bioRxiv 2021.08.26.457865

[CR144] Zhang L, Köhler S, Rillo-Bohn R, Dernburg AF (2018). A compartmentalized signaling network mediates crossover control in meiosis. Elife.

[CR145] Zhang L, Liang Z, Hutchinson J, Kleckner N (2014). Crossover patterning by the beam-film model: analysis and implications. PLoS Genet.

[CR146] Zhang L, Pickering R, Murray B (1999). Direct measurement of recombination frequency in interspecific hybrids between Hordeum vulgare and H. bulbosum using genomic in situ hybridization. Heredity (edinb).

[CR147] Zhang L, Wang S, Yin S (2014). Topoisomerase II mediates meiotic crossover interference. Nature.

[CR148] Zhang X, Clarenz O, Cokus S (2007). Whole-genome analysis of histone H3 lysine 27 trimethylation in Arabidopsis. PLoS Biol.

[CR149] Zheng B, Chen X (2011). Dynamics of histone H3 lysine 27 trimethylation in plant development. Curr Opin Plant Biol.

[CR150] Zickler D, Kleckner N (1998). The leptotene-zygotene transition of meiosis. Annu Rev Genet.

[CR151] Zickler D, Kleckner N (1999). Integrating Structure and Function. Annu Rev Genet.

[CR152] Zickler D, Kleckner N (2015). Recombination, pairing, and synapsis of homologs during meiosis. Cold Spring Harb Perspect Biol.

[CR153] Ziolkowski PA, Berchowitz LE, Lambing C (2015). Juxtaposition of heterozygous and homozygous regions causes reciprocal crossover remodelling via interference during Arabidopsis meiosis. Elife.

[CR154] Ziolkowski PA, Underwood CJ, Lambing C (2017). Natural variation and dosage of the HEI10 meiotic E3 ligase control Arabidopsis crossover recombination. Genes Dev.

[CR155] Zuo W, Chen G, Gao Z (2021). Stage-resolved Hi-C analyses reveal meiotic chromosome organizational features influencing homolog alignment. Nat Commun.

